# Lived Experiences of Physiotherapists in Caring for People with Advanced Amyotrophic Lateral Sclerosis in Portugal: A Phenomenological Study

**DOI:** 10.3390/bs15040510

**Published:** 2025-04-10

**Authors:** Andreia Monteiro, Amira Mohammed Ali, Carlos Laranjeira

**Affiliations:** 1 School of Health Sciences, Polytechnic University of Leiria, Campus 2, Morro do Lena, Alto do Vieiro, Apartado 4137, 2411-901 Leiria, Portugal; 5230014@my.ipleiria.pt; 2 Integrated Continuing Care Unit of Manuel Fanha Vieira, Rua da Barroca 60, 2330-108 Entroncamento, Portugal; 3 Department of Psychiatric Nursing and Mental Health, Faculty of Nursing, Alexandria University, Smouha, Alexandria 21527, Egypt; amira.mohali@alexu.edu.eg; 4 Centre for Innovative Care and Health Technology (ciTechCare), Polytechnic University of Leiria, Campus 5, Rua das Olhalvas, 2414-016 Leiria, Portugal; 5 Comprehensive Health Research Centre (CHRC), University of Évora, 7000-801 Évora, Portugal

**Keywords:** neurodegenerative disease, amyotrophic lateral sclerosis, palliative care, physiotherapy, rehabilitation, qualitative study

## Abstract

Amyotrophic lateral sclerosis (ALS) is a disease that has a multidimensional impact on a person’s life, with symptoms associated with a significant loss of autonomy. Specialized palliative care (PC) should be provided early and throughout the course of the disease. Indeed, physiotherapists should be understood as integral members of the multidisciplinary team in PC, in the care and improvement of the quality of life of these people. This study aimed to describe the lived experience of physiotherapists in the context of intervention in people with advanced ALS and their families. Descriptive phenomenology was employed as a framework for conducting semi-structured interviews to reveal experiences. Sixteen physiotherapists who performed interventions on at least one person with advanced ALS in the last 2 years were included in the study. The study involved conducting semi-structured individual interviews, through the Zoom^®^ videoconferencing platform (version 6.4.3). Data were analyzed according to Giorgi’s five-stage approach and managed using webQDA software (Version 3.0, University of Aveiro, Aveiro, Portugal). The COREQ checklist was applied in the study. Participants were mostly female (n = 12) and aged between 26 and 55 years (M = 36.81; SD = 6.75). Four constituents were identified: (1) undulating course of a complex disease; (2) barriers to person-centered care; (3) enablers of person-centered care; (4) transition between curative and palliative care. The findings illustrate the multidimensional impact of the disease trajectory on the person and their family. This study highlights the need to invest in specialized training for physiotherapists, contributing to a person-centered PC practice with an impact on promoting comfort and quality of life.

## 1. Introduction

Amyotrophic lateral sclerosis (ALS) is a chronic and incurable neurodegenerative disease that involves the progressive degeneration of motor neurons in the motor cortex and lower motor neurons in the spinal cord and brainstem (Brotman et al., 2024; Goncharova et al., 2021; Mangal et al., 2024). In Europe, the incidence rate of ALS is 2–3 cases per 100,000 inhabitants and is considered a rare disease (Longinetti & Fang, 2019). On average, death occurs 3–5 years after disease onset (Brotman et al., 2024; Mangal et al., 2024; Tzeplaeff et al., 2023). Nearly half of the people with ALS have varying degrees of cognitive and/or behavioral impairment, and approximately 15% meet diagnostic criteria for frontotemporal dementia (Brotman et al., 2024; Ilieva et al., 2023).

Initially, ALS affects the limbs and presents as a clinical characteristic the dysfunction of the upper motor neuron, which causes symptoms such as muscle weakness and spasticity (Erdmann et al., 2022; Meng et al., 2020; Zhu et al., 2022). When lower motor neuron dysfunction occurs, symptoms such as muscle cramps, fasciculations and atrophy appear (Longinetti & Fang, 2019; Meng et al., 2020). One third of cases have a bulbar onset, presenting with dysarthria and dysphagia and, later, with limb involvement (Longinetti & Fang, 2019; Meng et al., 2020). Sialorrhea is also a very common symptom and can cause several negative effects, such as skin maceration and exacerbation of dysarthria, which can result in increased psychological burden and social embarrassment (Wang et al., 2022). Respiratory symptoms, especially dyspnea, are common as the disease progresses; aspiration pneumonia and respiratory failure are the most frequent causes of death (Brotman et al., 2024; Filipe et al., 2024; Longinetti & Fang, 2019). Fatigue is also frequently reported by individuals with ALS (Alencar et al., 2022; Silva et al., 2024; Zhu et al., 2022). Additionally, as the disease progresses, depression, anxiety, and hopelessness are also commonly reported by people with ALS and their caregivers (Brotman et al., 2024; Heidari et al., 2021; Pagnini et al., 2022).

ALS is a disease that does not cause pain in itself, but this can occur mainly as a secondary complication of musculoskeletal dysfunction due to immobility, decreased joint range of motion, and difficulty in positioning in a wheelchair or in bed (Saavedra et al., 2020; Van Damme et al., 2024). Pain must be treated according to its cause by a multidisciplinary team, as it can negatively impact a person’s life on a physical, psychological, social, and spiritual level (Dal Bello-Haas, 2018; Hurwitz et al., 2021).

Guidelines recommend that specialist palliative care should be involved early and throughout the course of the illness, allowing the person to plan and discuss their care plan in advance (Cheng et al., 2024; Oliver, 2019; Shoesmith et al., 2020; Silva et al., 2024). With progressive muscle weakness, early and careful discussion of respiratory care, such as tracheostomy and ventilatory support, as well as the use of alternative feeding routes, is necessary (Brotman et al., 2024; Shoesmith et al., 2020). Clinical treatment is complex and requires a multidisciplinary approach (Andersen et al., 2012; Brotman et al., 2024; Shoesmith et al., 2020; Silva et al., 2024). Therefore, recognizing physiotherapists as integral elements of the multidisciplinary team in PC, their intervention must be adapted to the needs and objectives of the individual and family (Gonçalves & Magalhães, 2022; Silva et al., 2024; Wilson et al., 2022), as indicated by the WHO European Region document Policy Brief on Integrating Rehabilitation into Palliative Care Services (World Health Organization, 2023). As functionality is an important component of physiotherapy intervention, its main objective is to prevent and compensate for the decline in functionality, by optimizing mobility, function, and quality of life, through the prevention and relief of symptoms, and by teaching and training skills significant for the person, caregivers, and team (Silva et al., 2024; World Health Organization, 2023).

To assist in decision-making, with regard to physiotherapy assessment, three phases of disease progression are described: early/initial phase—despite muscle weakness in specific groups, people retain a greater degree of independence and mobility, with non-existent or minimal restrictions upon activity and participation (Dal Bello-Haas, 2018; Hoxhaj et al., 2023); intermediate phase—the number and severity of activity limitations increase due to disease progression, and restrictions on social participation are common (Dal Bello-Haas, 2018; Hoxhaj et al., 2023); late stage—individuals become totally dependent in mobility and function due to severe weakness of the axial and extremity muscles. Dysarthria, dysphagia, and respiratory problems are more common at this stage, although they can be present at any time (Dal Bello-Haas, 2018; Hoxhaj et al., 2023).

Late diagnosis makes it difficult to treat people with ALS during the early stages of the disease; however, starting therapy early can help delay the neurodegenerative process (Liu et al., 2019; Rosenbohm et al., 2023; Tzeplaeff et al., 2023). Therefore, physiotherapy plays a fundamental role in treating symptoms and maximizing function, thereby improving the quality of life of these people (Cheng et al., 2024; Dal Bello-Haas, 2018). Stretching, joint mobilization, and massage are recommended in the initial phase of the disease, as they are beneficial in preventing painful and function-limiting contractures and in reducing spasticity (Andersen et al., 2012; Shoesmith et al., 2020; Van Damme et al., 2024). Therapeutic physical exercise can help delay muscle deterioration in people with ALS (Cheng et al., 2024; Ortega-Hombrados et al., 2021; Shoesmith et al., 2020). This should be correctly monitored by the physiotherapist, always respecting signs of fatigue (Hoxhaj et al., 2023). Bronchial hygiene techniques, effective cough training, and hyperinflation techniques with Ambu bags are also recommended in this population (Saavedra et al., 2020; Van Damme et al., 2024), as well as the use of Cough Assist in more advanced stages of the disease (Saavedra et al., 2020; Van Damme et al., 2024). Inspiratory muscle training can improve respiratory function and heart rate in people with ALS (Vicente-Campos et al., 2022). In parallel, Sales de Campos et al. (2023) report that non-invasive ventilation and respiratory strength training can be effective, together with pharmacological approaches, in stabilizing respiratory function in ALS.

Hogden et al. (2017) recognize that the absence of multidisciplinary protocols that guide practice hinders communication between health professionals. They also reinforce the emotional burden that healthcare professionals face when caring for people with ALS, highlighting compassion fatigue and the moral and ethical challenges related to providing end-of-life care (Hogden et al., 2017). Olesen et al. (2022) report that healthcare professionals have difficulty balancing professional relationships with care team members and providing adequate support to caregivers/families. Health professionals also report difficulty in responding appropriately to the complexity and constant progression of ALS, influencing the quality of the response to the needs of the person and their caregivers (Olesen et al., 2022). The available evidence indicates the existence of educational needs for professionals in caring for people with ALS at the end of life and identifies the need for more resources and the reorganization of health services (Daneau et al., 2023). Professionals also reveal difficulty in emotionally engaging in monitoring people diagnosed with a progressive disease and with no prognosis for a cure (Walls et al., 2025).

Physiotherapists have a fundamental role in PC aimed at people with ALS, whether in educating caregivers/family and addressing emotional, cultural, religious, and spiritual issues, or in managing expectations and prioritizing desires and dignity in the advanced stages of the disease (Wilson et al., 2022). However, globally, rehabilitative PC practices are still not well understood, and service provision remains under-resourced and highly variable (World Health Organization, 2023). Therefore, one needs to understand the importance of these professionals, as well as their main barriers and enablers, when approaching people with advanced stage ALS. As far as we know, no studies on this topic have been carried out in Portugal, which follows a multi-sectoral PC model (including both home-based care with hospital care) characterized by high rationalization of resources and financial restrictions (da Cruz & Nunes, 2016). In this sense, a more in-depth knowledge of these experiences could contribute to greater specificity of the intervention, resulting in gains in the comfort and quality of life of these people. Thus, the main research question of this study is “What is the meaning and essence of the experience of care offered by physiotherapists in the context of intervention in people with advanced-stage ALS?” Therefore, this study aimed to describe the lived experience of physiotherapists in the context of caring for people with advanced ALS and their families.

## 2. Materials and Methods

### 2.1. Study Design

A qualitative study approach with a descriptive phenomenological design was adopted based upon the work of Amadeo Giorgi (2009, 2020). The phenomenological approach seeks to obtain rich data about lived experiences and perform a thorough phenomenological analysis of the data, with the perspective of phenomenological reduction (Willig et al., 2017). The use of this approach is related to the need to expand knowledge about lived experiences, allowing the researcher to answer the research questions and satisfy the study objectives. This approach presupposes a humanistic and ideographic perspective, as it studies the individual’s subjective experience (Englander & Morley, 2023), where meanings are the purpose or value people attribute to their actions or experiences (Davidsen, 2013; Englander & Morley, 2023).

The study followed the COnsolidated criteria for REporting Qualitative research (COREQ) checklist (Buus & Perron, 2020) (Supplementary Table S1).

### 2.2. Participants and Recruitment

The study involved Portuguese physiotherapists providing care to people with ALS and their families, either in a hospital or home setting. A purposive sampling method was used, based on the clinical experience of physiotherapists caring for people with advanced ALS. For the recruitment process, the following inclusion criteria were established: (1) having worked clinically as a physiotherapist for at least one year; and (2) having performed an intervention on at least one person with advanced stage ALS in the last 2 years. Participants who presented language barriers (speaking and understanding Portuguese), or who were absent from work due to vacation, sick leave, or others, were excluded.

The sample size was not defined a priori, and the data saturation criterion was used (Vasileiou et al., 2018). Data saturation was considered reached when the interviews with the participants no longer produced any new themes. Given that ALS is a rare disease, it is understandable that the number of physiotherapists with experience in intervening in people with advanced ALS is also reduced. Very specific samples require participants with very homogeneous characteristics, which can lead to smaller samples (Vasileiou et al., 2018). However, no limits were established in terms of gender, age, or professional experience of participants, allowing for maximum sample variation.

### 2.3. Data Collection

Initially, a purposive sampling method was used. The Portuguese Amyotrophic Lateral Sclerosis Association (APELA) was contacted and disseminated the study through its network of associates. Emails were also sent nationwide to palliative care teams that included physiotherapists and Integrated Continuing Care Units. Subsequently, snowball sampling was used, as participants who showed interest in participating publicized the study with their professional colleagues who met the inclusion criteria. Potential participants contacted the main researcher (A.M.) if they expressed interest in participating in the study.

Participants were presented with a brief description of the study, i.e., objectives, procedures, estimated interview time and general characteristics of participation. After completing the informed consent form, interviews were scheduled depending on the availability of participants and the interviewer.

Data collection took place between October 2024 and January 2025. To carry out this study, the researcher collected the participants’ sociodemographic and professional data to document their profile. The researcher (A.M.) conducted semi-structured individual interviews, using the Zoom^®^ video-conferencing software (version 6.4.3). The Zoom^®^ platform is recognized as a tool for collecting qualitative data due to its relative ease of use, cost–benefit ratio, data management capabilities, and security options (Archibald et al., 2019; Oliffe et al., 2021). It is worth noting that the use of telematics to conduct the interview widened coverage to a larger geographic area, sped up the scheduling process, and, consequently, allowed interviews with physiotherapists from different parts of the country (Archibald et al., 2019; Oliffe et al., 2021). A reflective diary was used by the researcher, aiming to record reflections after each interview. Participants were encouraged to remain in a calm place, without external interference and without the presence of other individuals who could disturb or influence the answers during the interview.

The interview script was based on the study objectives and supported by available evidence (Daneau et al., 2023; Walls et al., 2025; Wilson et al., 2022). The script comprised four domains: (1) the biopsychosocial and spiritual needs of the person with ALS and their family; (2) ethical and legal aspects implicit in care; (3) communication and teamwork; and finally, (4) the personal and professional issues that emerge from caring for the person with ALS and their family.

To verify the suitability of the interview, in terms of form and content, two pilot interviews were carried out with physiotherapists with characteristics similar to the sample. This allowed the identification of limitations in terms of comprehensibility, coherence, language, and order of the questions, allowing for review and the necessary changes to be made (Guazi, 2021). These pilot interviews were integrated into the sample, as no changes were suggested.

Each participant was invited to share their personal narrative, through the following open-ended question: “Tell me about your experience caring for a person with advanced ALS?” Follow-up questions were used as prompts during the interview, such as “What was important? What helped the most? What was difficult?”

The interview was conducted by the lead author, who has previous experience in qualitative interviews. She identifies as a cisgender woman and has eight years of experience in physiotherapy, particularly in intervening with people with advanced ALS. After the interviews were transcribed, they were returned by email to each of the participants so that they could analyze and verify whether the data collected correctly reflected their beliefs. The interviews lasted between 30 and 50 min (average 40 min). Only one interview was conducted per participant, and data collection was parallel to data analysis. After thirteen interviews, the researchers agreed that data saturation had been reached, as no new perspectives were presented on the main themes identified. However, the investigation was extended with three more interviews to ensure data saturation.

### 2.4. Data Analysis

Data analysis was based on the method proposed by Giorgi (Englander & Morley, 2023; Willig et al., 2017), divided into the five stages described in Table 1. Data analysis was performed using the qualitative data analysis software WebQDA (Version 3.0, University of Aveiro, Aveiro, Portugal). The authors coded all interviews, and discussions among researchers produced a consensus. The essential structure of the lived experience was organized into constituents and supported by fragments obtained in the interviews. Only after the analysis process were the textual quotes translated into English, maintaining their original meaning.

### 2.5. Trustworthiness and Reflexivity

The study strictly followed the eight criteria proposed by Tracy (2010), namely: “(1) worthy topic, (2) rich rigor, (3) sincerity, (4) credibility, (5) resonance, (6) significant contribution, (7) ethics, and (8) meaningful coherence” (p. 839). Regarding the worthy topic, the study is relevant and timely, since knowledge of the experience lived by physiotherapists in the context of intervention in people with advanced ALS may contribute to physiotherapy focused on comfort. The rigor of the study was guaranteed by following the procedures established for data collection and analysis, optimizing the quality of the findings. The transcripts of the interviews were sent to each of the participants so that they could analyze whether the collected data correctly reflected their beliefs. The sample size was defined when data saturation was reached (Johnson et al., 2020). Sincerity was achieved through self-reflexivity, vulnerability, and transparency in data collection. The researcher engaged in moments of introspection, evaluating their own biases and motivations (phenomenological reduction). Only the first and last authors (A.M. and C.L.) have experience of caring for people with neurodegenerative diseases and are familiar with the research subject. In parallel, two authors (A.M.A. and C.L.) hold a PhD degree and have previous experience in conducting qualitative studies. Credibility refers to the reliability, verisimilitude, and plausibility of the research results (Tracy, 2010). To this end, the triangulation of researchers was used to foster credibility and, consequently, the rigor of the qualitative study (Johnson et al., 2020). In addition to conducting individual interviews, the main researcher (A.M.) wrote a diary with their reflections after each interview and took field notes on contextual details and non-verbal expressions, thereby optimizing accuracy in the data collection and analysis processes. To achieve resonance, the study methodology was thoroughly described and schematically represented, promoting its transferability to similar contexts (Johnson et al., 2020). This study also followed the criterion of significant contribution, since there are no studies on the subject in Portugal, thus contributing to greater specificity of physiotherapy intervention in people with advanced ALS. All procedural and relational ethics procedures were followed to recognize and value mutual respect, dignity, and connection between the researcher and participants. Finally, meaningful coherence was achieved through an interconnection between the literature review, the methodology, and the obtained findings.

### 2.6. Ethics

This study was approved by the Local Ethics Committee (CE/IPLEIRIA/86/2024) and followed the principles of the Declaration of Helsinki. The purpose of the study was duly explained to all participants, and the anonymity and preservation of their reports were ensured. The researcher informed the participants that the interview could be interrupted at any time, without any associated consequences. Informed consent was made available to all participants. The recordings of the interviews were stored in a file that only the researcher had access to via password and, subsequently, will be destroyed one year after the project’s conclusion. There was no financial compensation for participating in the study.

## 3. Results

### 3.1. Sample Description

Sixteen physiotherapists participated in the study, most of whom were female (n = 12) and aged between 26 and 55 years (M = 36.81; SD = 6.75) (Table 2). Participants had between 4 and 34 years of professional experience (M = 14.44; SD = 7.1). Regarding academic qualifications, nine participants had a bachelor’s degree, and the rest had a master’s degree (n = 7). Half of the participants reported not having specific training in palliative care (n = 8). The majority worked in a community/home context (n = 9), followed by specialized rehabilitation units (n = 7), and finally, in specialized palliative care units (n = 2).

### 3.2. Findings from Interviews

Data analysis provided a detailed understanding of the lived experience of physiotherapists in intervening with people with advanced ALS. As a result of the analysis of the interviews, four constituents and fifteen subconstituents were identified (Table 3).

#### 3.2.1. Constituent 1: Undulating Course of a Complex Disease

Constituent 1 is divided into two subconstituents: (a) rapid decline and decreased functionality and (b) non-acceptance of the disease and unrealistic expectations of a cure. All participants experienced specific worries regarding the suffering of ALS patients required them to explore the complexity of patients’ healthcare problems and illness situations to discern the level of required care.

(a) Rapid decline and decreased functionality

Physiotherapists describe the course of the disease by addressing the physical and functional changes associated with ALS, from the onset of the first symptoms to the advanced phase characterized by total functional dependence. In most cases, the disease has a rapid and overwhelming progression, characterized by ups and downs, significantly impacting quality of life and leading to early and sudden death.

*P1: It was galloping, it was very fast, maybe one of the fastest situations I’ve ever experienced. Because it took her a year to try to confirm the diagnosis and then (…) she died a maximum of two years after the diagnosis.*

*P12: He entered the unit walking with a walker, but for very short distances. (…) He was about 50 years old. (…) He already had a lot of difficulty, but he still spoke and ate orally. When he left, he literally only moved his eyes. He already had a gastrostomy (PEG), He couldn’t speak anymore, and used an alternative communication system.*

*P15: It started with hoarseness and some muscle weakness. He learned of the diagnosis at the age of 44 and then died at the age of 46, with the disease progressing rapidly.*

Symptoms, in general, vary according to the reported cases and the phenotype of the disease, including motor and functional changes, pain, respiratory impairment, dysphagia, and communication difficulties.

*P3: ALS is a disease that does not normally cause pain, (…) but immobility, rigidity, spasticity, positioning, all of this causes pain. Therefore, pain is a physical symptom that is always present. Some of them even have some difficulties speaking (…) and difficulty swallowing (…).*

*P7: The loss of mobility, changes in sensitivity and the respiratory part above all. This gentleman’s situation was bulbar ALS. The respiratory part was the one that was most affected. There were several respiratory changes, breathing difficulties, changes in breathing pattern, swallowing changes, movement changes.*

*P14: Decline in the hands… The hands immediately catch our attention in our practice because they are patients who are already ventilated and who need to have some autonomy to put on/take off the mask, turn on/off the device. Not to mention the basic activities of daily living, dressing/undressing, etc. (…) they easily lose the ability to communicate.*

(b) Non-acceptance of the disease and unrealistic expectations of a cure

The rapid progression of the disease creates difficulties in accepting the disease and its functional consequences, given that most of the time this pathology does not cause cognitive impairment. Considering the mismatch of the person’s expectations with advanced ALS (but also by their families/caregivers), there are cases of anger that lead to non-adherence to treatment.

*P3: He refused to use the ventilator at night, even though he was aware of the risks he was running because we explained them to him… I think that deep down he also planned the evolution of the disease. (…) This revolt ended up being the end for him. The last hours of his life were filled with suffering.*

*P5: Refused the ventilator and all support measures (…) refused Palliative Care.*

*P14: (…) sometimes we are confronted with people who are not yet fully aware, both the patient and the family, of what the progression of the disease will be like. We are often faced with, “Is the ventilator for use? Is it for continued use or is it just until I get better?” Or, for example, “Oh, but is this cough machine for everyday use, or just once in a while?*

There are also situations when the family does not accept the condition and has unrealistic expectations regarding the person with ALS and the need for physical therapy.

*P4: The biggest difficulty is the family’s management of the patient. I often see families who exert a lot of pressure on the patient or on the team to carry out their convictions.*

*P15: In fact, he (the patient) had accepted the disease more than the family. The family was never satisfied because they did not want him to die, and they really did not accept the progression of the disease.*

*P9: And sometimes it’s very difficult and very frustrating to try to convey that idea to caregivers. For example, I go there twice a week, and they still pay more for private services. Because you can’t stand still. And this does not reverse the process of that descending phase.*

#### 3.2.2. Constituent 2: Barriers to Person-Centered Care

Constituent 2 is divided into six subconstituents: (a) lack of intra- and inter-professional communication; (b) late diagnosis and referral to PC; (c) shortage of human and technical resources; (d) lack of specific training; (e) caregiver burden; and (f) precarious socioeconomic conditions of families.

(a) Lack of intra and interprofessional communication

Participants describe difficulties in communication within the multidisciplinary team during their work. P9 points to the lack or absence of organization and method in team meetings, contributing to the inadequate or insufficient discussion of cases: *In fact, we don’t even discuss users as we should. We have a lot to do in terms of teamwork. There are meetings among professionals, or they are very rare. When they exist, they are just conversations, but they are not very structured (…) there is a lack of method.*

Furthermore, P4 states that the quality of the information transmitted or discussed in meetings depends on the perception of the team members present: *I think communication has flaws, as it depends a lot on the perception of those present. The doctor, myself, the social worker and the psychologist are always the same. However, nurses and assistants rotate and it depends a lot on each person’s profile. There are those team members who are more human and who truly value the patient’s entire environment (…). There are others who are very focused on the task part and discard everything else.* P4 also adds that loss of information transmitted during meetings is a communication barrier, expressing concerns about this problem: *the meeting is on Tuesday and certain information is passed on, but on Thursday or Friday, this information has already been lost during the shift changes.*

At the same time, participants report that there is difficulty among health professionals in sharing knowledge, given the existing competitiveness and the difficulty in recognizing weaknesses.

*P8: We have a lot of difficulty in sharing, in talking, for example, about failures, about what works, what doesn’t work (…) I think that in our area (physiotherapy) we would have a lot to gain if we shared more (…) This would allow us, especially in rare diseases, to feel more supported in decision-making.*

*P16: I think it is really closely associated with an inability to communicate between professionals. There are a lot of egos among healthcare professionals, and that’s why things don’t work out. The physiotherapist is also often not seen as an active voice.*

The fact that there is no real teamwork between healthcare professionals from the different specialties who treat people with ALS was also addressed, with reference to communication failures or even difficulties in ensuring a safe transition of care.

*P15: I really think there is a lack of a cohesive team, true teamwork (…). A multidisciplinary team that is truly there to support these people and their caregivers, because they feel unsupported and pushed from one specialty to another.*

*P1: There is no communication between the hospital and the health center. They refer the user to us, we have access to generic information about the process and when people go to the consultation, they bring their consultation notes. But we don’t communicate directly, which I think is a big flaw.*

*P9: Even with palliative care itself… there is not much communication with us. There is still, in general, a lack of coordination between primary health care and hospital care… which leads to a fracture in care.*

At the same time, difficulties in communication between professionals generate conflicts and disagreements between team members, impacting on the quality of services provided to people with ALS.

*P8: There is conflict between teams, multidisciplinarity does not work in managing this disease.*

*P10: I currently have a situation where a user was referred to another hospital for percutaneous endoscopic gastrostomy (PEG) placement. This is because the doctor at the hospital where he is being treated said he would not be able to handle the placement of a PEG, having opted to place a nasogastric tube to stabilize his weight and only then consider the PEG. Deciding to place a PEG is to minimize patient suffering, and any decision must be coordinated between teams.*

(b) Late diagnosis and referral to PC

Participants say that late diagnosis works as a barrier to their intervention, limiting access to specialized intervention targeted at people’s real needs. Often, ALS symptoms are confused with other musculoskeletal or cardiorespiratory conditions, and patients are referred to physiotherapy in an undifferentiated manner.

*P11: A gentleman who entered the unit with a diagnosis of stroke, without image translation, (…) later the diagnosis was confirmed (ALS).*

*P9: He was referred to a diagnosis of peripheral polyneuropathy of the upper limb. And he spent months on this, until the diagnosis was confirmed.*

P15 claims that these conditions arrive at private *clinics disguised as some pain, with some functional limitation, when in fact it is not just a functional limitation, it is not just pain, it is much more than that*. P16 highlights the need to invest in tests for a correct and timely diagnosis, in order to speed up access to care targeted to the person’s needs. He also states the following: *if we listen to people and value what is said, perhaps we will gain more time and be able to provide a better quality of life*.

Participants also revealed that people with ALS are referred to PC late or, when it occurs, it is insufficient or inadequate. P9 states the following: *the palliative consultation often arrives too late. These users need help with their own adaptation to their health condition.* He also claims that the buck is passed around, subjecting people to bureaucratic processes to access care and generating feelings of frustration. He adds that *physiotherapy responses in the area of palliative care are very residual, and there is still a long way to go in this regard*. Two participants also mentioned that: *only the most advanced cases end up having palliatives* (P1); *only receive palliative care in the last week of life* (P3).

(c) Shortage of human and technical resources

Participants highlighted the difficulties health services have in recruiting physiotherapists, which hinders the response to the complex needs of people with advanced ALS and their families. This shortage of human resources is common in different contexts, which is why participants report being unable to closely monitor these cases.

*P5: The fact that I am the only physiotherapist on the team sometimes complicates the monitoring of these patients. More physiotherapists are needed in teams and in homes.*

*P9: Users are always joining; I feel a lot of pressure to add new users and to release them. So basically, I’m just running around. And I don’t spend enough time with these users, which I think I should.*

*P12: Given the number of professionals, it was becoming increasingly complicated to manage their needs. So, the doctor requested a transfer to another service, so we could try to meet his needs. He especially needed more time from us.*

Participants highlight that the lack of physiotherapists in the services means that they are not always present at family meetings/conferences in which diagnosis, the integrated care plan, and the prognosis are discussed.

*P4: I just attended a family conference for an ALS patient. I have a very small number of hours allocated to the Palliative Care unit. It’s three hours a day and there’s not enough time for everything (…).*

*P6: Family conferences are held, but they’re mostly with the doctor, nurse, social worker and psychologist. The physiotherapist usually doesn’t come in.*

*P11: We were not always present at family meetings, because we need to manage activities, and we are few professionals.*

At the same time, the limited availability of technical assistance in responding to the needs of patients and family members was also mentioned. P1 mentions the following: *There is not always an articulated bed, a transfer lift or a suitable armchair.* Given these limitations, P15 refers to *the importance of faster responses from the Unified Health System.* As the disease progresses, the movement of a person with ALS becomes difficult, requiring a lot of logistics and appropriate transport. Existing restrictions limit access to physiotherapy and exacerbate the risk of psychosocial isolation.

*P3: At an advanced stage it is necessary to have appropriate transportation for the person to come to us. And often this transportation does not exist. There is a co-participation from Social Security in transportation, but sometimes the person has to come up with the money and people do not have that possibility.*

*P8: We lose the ability to care for a patient, not because he is physically weakened by the disease, but because we do not have the ability to compensate for his limitations, which are social, transportation, access and taking him out of the house. A patient stays here for an hour or two and likes to come and socialize.*

(d) Lack of specific training

Participants indicate a lack of specific training in PC as a barrier to the provision of quality care. Therefore, curricula must be redesigned to prepare physiotherapists for an approach that increases comfort, meets physical, psychological, social, and spiritual needs, that is, a person-centered care practice.

*P6: We are often prepared to rehabilitate (…) when our basic training in this type of context (palliative care) does not exist.*

*P15: In my initial training in physiotherapy, we didn’t talk about PC at all. In fact, many physiotherapists do not know what palliative care is because it is not discussed.*

The training gap affects all members of the multidisciplinary team and delays the referral process for specialized PC. Continuing education programs provided by employers generally do not include the topics of palliative care and bereavement.

*P9: There is a lack of training. Employers offer little training and when they do, they show little interest. (…) There is a lack of training on palliative care and end-of-life communication.*

*P4: On a personal level, I feel that I need to develop myself a little more, to have better strategies and skills to deal with these patients and caregivers, as well as in managing expectations and grief.*

(e) Caregiver burden

Participants identified the existence of stressful situations and caregiver exhaustion, as people with advanced ALS require permanent and highly complex care. Caregivers neglect their own needs in favor of caring for the person with ALS. This burden has a significant impact on the provision of care to people with ALS, influencing the ability of caregivers to deal with situations of exhaustion.

*P14: There are caregivers who are isolated, after all, they are the ones who take care of the sick all the time. They feel this pressure and become physically and emotionally exhausted.*

*P3: I remember a wife who was very tired because every night was a night of screaming, a night of despair, he wouldn’t let her sleep, because he was afraid of the night.*

(f) Poor socioeconomic conditions of families

The socioeconomic frailty of families is a reality, limiting access to therapies and support products. Families cannot stop working since there is no financial support, preventing people with advanced ALS from remaining with their families for longer.

*P9: We really need to have the working caregiver status… because in reality it doesn’t work… One of the patients you see has a wife who works. I had the feeling that she was having immense difficulty managing everything: work, supporting her husband, going to physiotherapy.*

*P12: It would be easier if we had more social responses to care. I believe that some families wanted to keep patients within the family for longer, but they ended up having to institutionalize the patients.*

*P15: There is a lack of financial resources, there are expensive support products that family members could not afford. Some could only continue with the therapies because they had some savings.*

#### 3.2.3. Constituent 3: Enablers of Person-Centered Care

Constituent 3 is divided into three subconstituents: (a) collaborative teamwork; (b) specialized support from associations supporting people with ALS; and (c) therapeutic relationship with ill person and family.

(a) Collaborative teamwork

Teamwork should form the basis of intervention in people with advanced ALS in order to respond appropriately to their needs. Sharing knowledge and holding joint sessions are an added value and allow teams to offer services focused on the needs and preferences of these people.

*P2: There was a woman with ALS who was admitted to continuing care as an inpatient. The team was not very familiar with Cough Assist (a device that stimulates the elimination of phlegm). I went to help, after all it is important that we can help our colleagues.*

*P10: We developed work together, that is, if the physiotherapist had a relationship of trust with the patient, the psychologist would take advantage of this relationship of trust and then the work would be done together, which would allow the psychologist to be better informed about the situations and to act more effectively.*

(b) Specialized support from associations supporting people with ALS

The collaborative and specialized work offered by associations supporting people with ALS is recognized by participants as a facilitator for both the person and family and for the teams working with these users. Teams find advice and updated information in these associations on how to intervene appropriately.

*P3: A very big facilitator that I think we have in the association is the support product bank, which basically provides support products when the person does not yet have their own.*

*P12: I think the association makes it much easier for us to access this information.*

*P14: When caring for these patients, we always refer ALS patients to the association… for the differentiated support and all the information they can provide to improve the quality of life of users and their families. The association’s own colleagues try to talk to us.*

Two participants highlighted the association’s fundamental role in meeting the needs of the national health system, carrying out complementary work in supporting people with ALS and families/caregivers. In this regard, P8 states that *the work of the association is complementary work and can never replace the health system.* P9 adds that *the association helps in the response that the State is unable to provide. Whether it is in the way of communicating with users, making them better integrate the issue of diagnosis and prognosis, and also social responses.*

(c) Therapeutic relationship with ill person and family

Participants highlight the importance of establishing a close therapeutic relationship with the person with advanced ALS and family/caregivers, based on empathy, trust, and the availability to help clarify doubts whenever necessary.

*P5: I think they feel understood and trust us, it’s a privilege. It is a relationship of trust with the person and the family. A relationship is created with these people.*

*P14: A facilitator is us being available. Families recognize this. I show my willingness to make things go as smoothly as possible.*

*P16: And, essentially, having a good relationship between the physiotherapist and the user. I think it has to be based on empathy, on humor.*

From the participants’ perspective, the family/caregivers constitute a facilitating element in the care of the person with advanced ALS, as they best understand the person’s particularities, thereby facilitating communication and the therapeutic relationship. Furthermore, in a home context, the family/caregiver is someone who provides care and is with the person on a daily basis. They are generally open to receiving teachings and guidance from physiotherapists.

*P9: The great facilitators are usually the caregivers. They are available, always receptive to our teachings and guidance.*

*P15: Family members are usually facilitators of care, because they are the ones who understand everything that patients need.*

*P16: Family and caregivers end up being that bridge that connects us to the user. Sometimes we have difficulty communicating with patients and the family ends up giving us some tips on how to approach the person.*

#### 3.2.4. Constituent 4: Transition Between Curative and Palliative Care

Constituent 4 is divided into four subconstituents: (a) access to narratives as a strategy for preserving human dignity; (b) respect for wishes and preferences; (c) seeking palliative care physiotherapy; and (d) offering psychosocial and spiritual support.

(a) Access to narratives as a strategy for preserving human dignity

Through narratives, participants access the world of life and recognize the importance of adapting their interventions to provide well-being. P13 addresses the need to assume comfort as the core of intervention in people with advanced ALS. P13 verbalizes the following: *what makes the most sense during this period is comfort and quality of life. The little gestures and the little details are so important. However, we are sometimes only focused on rehabilitating.*

Physiotherapy intervention is also described by participants as preserving dignity, whenever it provides users with opportunities to experience pleasant sensations and even fulfill last wishes. P14 says: *He* (patient) *and his family really wanted to go for a ride on a tricycle-type vehicle with friends. For him to go we needed to have a support on the seat, stabilization so as not to lose control of the trunk, a battery-powered fan (…). We tried to help and be an active part, to help him with something that was important to him.*

In this regard, P7 praises physiotherapists, considering them to be fundamental elements, given that they are support figures for people with advanced ALS, stating that *in addition to being physiotherapists, we are human and we maintain the dignity of the person and can use our strategies and resources to help minimize suffering. The benefits of physiotherapy for people with advanced ALS and their families go far beyond the implementation of rehabilitation techniques, but also include spiritual and emotional benefits.* P3 says: *Sometimes I felt that maybe it didn’t make much sense for us to be intervening in that case anymore, but at the same time, when she went there, I always felt the opposite, because she was so happy. Although there were no benefits in terms of physical therapy, the effects were on another level, in terms of spiritual and emotional well-being.*

(b) Respect for wishes and preferences

As the disease progresses, people with ALS and their families face difficulties in making decisions. Participants described that they are often sought out to clarify doubts regarding end-of-life decision-making. In this sense, P8 mentions that *it is up to the patient to make informed choices, we are here to work and help them make informed decisions. If they choose not to be mechanically ventilated, they will not be.*

Regarding access to living wills, most participants claim there is still a great lack of knowledge on the subject among people with ALS and their families. P9 also mentions the persistence of a very paternalistic view in relation to healthcare, *so people tend to do what doctors say*, a reflection of the population’s characteristics in relation to health and death issues. P16 adds the need to introduce the living will earlier, that is, *while the person still has the capacity to decide*. P10 states that there is a lack of structure in accessing the living will, that is, *an individual care plan is not actually made (…) it depends on where the professional is facing and whether they remembered to address the issue at that time or not.* He also adds that there is no health professional responsible for doing this: *sometimes it comes from the family doctor, other times it comes from the neurologist in the hospital, other times, in more advanced stages, with the palliative care team that goes to the home.* P8 highlights the importance of informing people about issues related to advance directives: *People have to be well informed about the issues related to advance directives, step by step*. However, P3 says that there is an increasing demand for information on this topic from the association: *(…) even our social worker colleague tells us a lot that people already ask about them. They want to know how it works, how they can do it. And I think that’s a very good thing, don’t you? We are changing the way we see things here now and I think that is important.*

(c) Seeking palliative care physiotherapy

The fine line that separates the curative and palliative approaches is something that worries participants. They describe the difficulty in initiating a palliative approach, given that medical complications are constant in ALS, and they recognize that this does not depend on their work, but that it is sometimes difficult to manage emotionally. P9 mentions the following: *There is a bit of frustration because, in fact, with these users our intervention is only to try to minimize a progression that will happen and that has much more to do with other intrinsic factors of the user and their specific disease than with our intervention itself. So, we like to feel like we’re in control of the process and we’re not. The feeling is of a total lack of control over the process, we don’t know if the disease will progress faster or slower.*

Participants recognize the importance of consolidating PC physiotherapy in symptom control, maximizing functionality, improving quality of life and preventing complications.

*P3: Promote passive mobilization of limbs, stretching to prevent further spasticity or shortening of limbs due to immobility. Prevent joints from becoming stiff and causing even more pain. Touch and massage are strategies that we use a lot when someone is suffering.*

*P4: I always try to enhance the strength that exists, associated with some function that is relevant in people’s daily lives. So, firstly, it is about enhancing, optimizing what exists, and secondly, preventing complications.*

*P13: I invest in mobilization to provide some comfort, despite already having some deformities and being bedridden for a long time.*

Finally, P12 refers to the need to recognize signs of fatigue and manage the intervention according to tolerance: *we must prioritize passive mobilization and non-fatigue*.

(d) Providing psychosocial and spiritual support

Participants report that caregivers often neglect their own needs in favor of caring for the person with ALS. P9 says *I think they (caregivers) see that they have that mission*. P10 adds the following: *I followed a caregiver of about 29 years old, who suffered from burnout and assumed that she was failing in her care due to exhaustion. In this sense, participants consider that strengthening psychosocial and spiritual support for both people with advanced ALS and caregivers constitutes a strategy aligned with the philosophy of palliative care.*

*P2: Sometimes caregivers are overwhelmed, and we have to make them see that they don’t have to cope alone. It is important that they know when to ask for psychological support.*

*P3: Grief starts in the beginning, from the first moment a person has ALS. Family members cry during the process of functional loss, and everything that the disease entails. That is why psychological and spiritual support is so important.*

During their speeches, participants reported encountering existential questions from people with advanced ALS, who share reflections on life, regrets, fear of finitude, and legacies.

*P4: In the advanced stages of the disease, people review their lives and have a lot of things they would love to have done differently. I intervened with a man who was angry about his illness, but at peace with his life. He wrote poems that he passed on to his children and wife. I know they were working on a publication at the time. The idea of leaving something written was a strategy to work on spiritual restlessness.*

*P3: The fear of death is not about death, but about the fear of leaving this world. It is the process of dying. Questions like: So how will the family cope without me here? Will I suffer?*

## 4. Discussion

To the best of our knowledge, this is the first phenomenological study in Portugal that explores the lived experience of physiotherapists in approaching people with advanced ALS. Therefore, this study offers an in-depth understanding of physiotherapists’ perspective of the impact of ALS on the person and family/caregivers, as well as aspects related to the complexity of care.

The present study highlights the undulating course of the disease trajectory, characterized by rapid progression and decline in autonomy and functionality of the person with ALS, with a significant impact on quality of life. According to the available evidence, the trajectory of ALS is characterized by a set of physical and functional changes with an emotional impact and on the well-being of the person with ALS and family/caregivers (Brizzi et al., 2020; Warrier et al., 2020; Yu et al., 2024). Studies also highlight the difficulty that people with neuromuscular diseases and their families have in constantly adapting to the challenges caused by these diseases, which involve rapid decline and constant changes in daily life (Handberg & Werlauff, 2023; Pinto et al., 2021). Studies underline the importance of professionals in establishing shared goals for the rehabilitation process, as well as providing structured support that includes psychosocial and spiritual support and helping to manage concerns about an unpredictable future (Handberg & Werlauff, 2023; Pinto et al., 2021). The present study also reveals that the non-acceptance of the disease can cause anger and frustration in people with ALS, which can affect their adherence to treatment. Loss of function or skills and lack of support from the health system can trigger feelings of anger, frustration or sadness (Glennie et al., 2023; Handberg & Werlauff, 2023; Pinto et al., 2021; Velaga et al., 2023). Adherence to treatment can be improved when people feel that professionals care about their emotional and physical needs (Annunziata et al., 2023).

One of the barriers to care arises from the late or incorrect diagnosis of ALS. A recent analysis of diagnostic trends in Türkiye, Germany, Poland, and Portugal reported a mean delay in ALS diagnosis of 11 months after symptom onset (Rosenbohm et al., 2023). Thus, incorrect diagnosis of ALS can result in delays in accessing appropriate treatment and in referral to palliative care. Therefore, it is crucial to improve the diagnostic pathway and promote greater awareness among people and health professionals of the signs and symptoms of ALS and the benefits of referral to multidisciplinary care (Vitorino et al., 2023; Goyal et al., 2024; Rosenbohm et al., 2023). Participants in this study also indicated the lack of intra- and interprofessional communication as a barrier to care, making coordination between health professionals difficult. In line with these findings, recent studies underline the importance of communication, effective collaboration and teamwork when approaching people with ALS (Parola et al., 2018; Walls et al., 2025; Wilson et al., 2022).

Also consistent with our findings, Zeng et al. (2025) reflects on the impact of functional loss on the life of people with ALS, their communication difficulties and the needs related to mobility and transportation, demonstrating the difficulties in accessing support products and traveling to appointments. Indeed, the use of assistive technology (support products) should be promoted in people with neurological conditions, improving their adaptability, competence, and self-esteem, and reducing participation limits (Jiménez Arberas et al., 2021).

Similarly, Chen et al. (2023) identify work overload as a barrier to receiving the benefits of PC in degenerative disease, as there is no time to communicate with people. The specific characteristics of the physiotherapist-patient relationship can influence the success of rehabilitation, and promoting integrated professional support for the body and mind is essential (Monaco et al., 2022). According to Miciak et al. (2018), there are conditions that facilitate the therapeutic relationship in physiotherapy: (a) being present; (b) being receptive and open to negotiating appropriate treatment plans; (c) being genuine; and (d) being committed to honoring the person’s interests. This therapeutic relationship means that physiotherapists are sometimes sought out to clarify doubts regarding decision-making, as reported in our study. Therefore, it is important to consider that person-centered care and decision-making are based on providing people with dignity, respect and compassion, and offering coordinated care and personalized service (Hogden & Crook, 2017; Laranjeira & Dourado, 2022; Laranjeira, 2023). Shared decision-making, whereby patients, caregivers, and health professionals collaborate to reach a care decision, is considered the best practice for delivering person-centered healthcare (Hogden & Crook, 2017; Thorborg et al., 2023). On top of this, by incorporating patients’ preferences, needs, and values into their practices, professionals can improve satisfaction in patients with ALS and families (Kierkegaard et al., 2021).

Furthermore, participants in our study identified caregiver burden as a barrier to caring for people with advanced ALS. Several studies report the levels of caregiver burden, which leads caregivers to neglect their individual needs, and the financial impact of ALS on families (Asano et al., 2021; Beyermann et al., 2023; Brizzi et al., 2020; Maksymowicz-Śliwińska et al., 2023). In a home context, the family/caregiver provides care and is with the person on a daily basis, therefore it is essential to educate them and provide guidance to ensure proper care (Wilson et al., 2022). Optimal home PC depends on close collaboration and dialogue between the ill person, family, and healthcare professionals (Danielsen et al., 2018).

In PC, physiotherapists play a fundamental role in the multidisciplinary team. They contribute to the identification of various needs throughout the disease trajectory, improving the functionality and quality of life of people and families (Wilson et al., 2022). When properly integrated into PC teams, physical therapy can aid in symptomatic control and help people remain in a safe home environment (Ogundunmade et al., 2024). However, recognition by professional organizations is necessary regarding the integration of more physiotherapists with specialized training in PC in these services (Ogundunmade et al., 2024; Wilson et al., 2022). The lack of training in PC among physiotherapists generates a lack of confidence in monitoring individuals with life-limiting illnesses and regarding their role in PC teams (Ogundunmade et al., 2024; Wilson et al., 2022).

In the present study, participants recognize difficulties in the transition from curative to palliative care, as well as in emotional management itself. Other studies report the lack of training and the emotional and moral suffering of physiotherapists working with a progressive disease with no potential for cure causes. Thus, the development of support and education systems that help health professionals in this emotional involvement is important (Monaco et al., 2022; Walls et al., 2025). Participants recognize the importance of physiotherapy in symptomatic control, maximizing functionality and improving the quality of life of these people, through techniques aimed at comfort and well-being. These findings are in line with the available evidence on physiotherapy in PC and ALS (Mangal et al., 2024; Ogundunmade et al., 2024; Saavedra et al., 2020; Sales de Campos et al., 2023; Shoesmith et al., 2020; Van Damme et al., 2024). Furthermore, our findings highlight that the main purpose of physiotherapy for people with advanced ALS should be meeting their physical needs, but also their psychological, social, and spiritual needs. Studies emphasize the promotion of comfort in PC through symptomatic control, compassionate care, connection to spiritual or religious practices, and the establishment of robust therapeutic relationships (Coelho et al., 2016; Matos et al., 2024; Timóteo et al., 2024; Wensley et al., 2020). Furthermore, evidence points to a set of sources of hope for people with ALS, namely: (a) preservation of dignity and control of decisions related to care; (b) family as a source of comfort and meaning in life; (c) valuing social interaction and support from professionals and services; (d) establishing realistic goals; (e) facilitated access to support products/assistive technologies; and, finally, (f) the search for meaning and purpose in life (Gonçalves et al., 2023; Pinto et al., 2021; Soundy & Condon, 2015).

### 4.1. Strengths and Limitations

This is a pioneering study in Portugal, as there are no known studies on the subject to date. It also contributes to the expansion of available knowledge regarding the lived experiences of physiotherapists in caring for people with advanced ALS. Despite the merit of the study, some limitations were identified. Firstly, it was difficult recruiting physiotherapists experienced with advanced ALS patients, given that it is a rare disease. Secondly, participants’ views may have been affected by their amount of experience caring for patients with ALS. Thirdly, the interviews were conducted via a video conferencing platform, which may have masked some emotional aspects and non-verbal language. Some challenges related to establishing calls, poor internet connection, audio quality, and environmental distractions were also identified. All these factors introduce bias that impacts the quality of findings and their transferability. Further research may be conducted with larger samples and in different cultural contexts to validate and broaden the findings. Additional efforts are required in clinical practice, research, and education to comprehend the intricacies of ALS.

### 4.2. Implications for Practice

This study seeks to advocate for a practice centered on the person and the biopsychosocial and spiritual needs of people with advanced ALS and their caregivers and families. It also contributes to a vision of physiotherapy focused on comfort, highlighting the physiotherapist’s role in PC and some strategies that allow the transition between curative and palliative care. The need to address the topic of PC in entry-level postgraduate physiotherapy curricula and continuing education is recognized, helping these professionals to base their actions on the principles of PC. Beyond physical advantages, physiotherapists may assist patients in optimizing and maintaining their dignity amidst variations or decline in their condition; this is achieved through direct therapeutic interventions, as well as facilitating communication, education, and collaboration among caregivers and the interdisciplinary team (Mercadante & Al-Husinat, 2023; Ogundunmade et al., 2024). In addition, physiotherapists may support decision-making processes and foster early advanced care planning which helps patients and caregivers to define goals and preferences for end-of-life care (Thorborg et al., 2023; Vandenbogaerde et al., 2022).

Integrating palliative care with primary healthcare services, including physiotherapy, could enhance access for patients residing in distant areas. This would facilitate access for patients and their families to complete care without the encumbrance of significant personal expenses (Danielsen et al., 2018). This additionally mitigates the possibility of incurring expensive institutionalizations or hospitalizations resulting from insufficient care provided at home. Finally, more studies are needed that reflect on the experience of physiotherapists in caring for people with ALS and their caregivers, as well as the creation of guidelines and good practices for PC physiotherapy, which requires more practice, confidence and resources.

## 5. Conclusions

This study illustrates the lived experience of physiotherapists in the context of intervention in people with advanced ALS, reflecting the multidimensional impact of the disease trajectory, as well as main barriers, facilitators to care and intervention strategies. The participants’ lived experiences were organized into four constituents: (1) undulating course of a complex disease; (2) barriers to person-centered care; (3) facilitators of person-centered care; and (4) transition between curative and palliative care. Thus, this study highlights the importance of PC physiotherapy in the care of people with advanced ALS, contributing to a person-centered practice, with an impact on promoting comfort and quality of life. On top of this, policy and clinical guidelines are urgently needed, supported by professional development programs. Likewise, the absence of referral criteria, guidelines, or tools delineating those who may benefit from physiotherapy in the context of PC restricts its availability.

## Figures and Tables

**Table 1:** Giorgi’s five-stage approach to data analysis (Englander & Morley, 2023; Willig et al., 2017).

**1st stage:** Reading the complete transcript of the interviews	The researcher begins by reading the descriptions several times in order to grasp the overall meaning of the lived experience by each participant.
**2nd stage:** Adoption of an attitude of phenomenological reduction (epoché)	Adopting an attitude of phenomenological reduction, putting aside knowledge about the phenomenon under study, so that it can present itself in its entirety.
**3rd stage:** Dividing data into meaning units	Rereading the descriptions to facilitate data analysis, breaking them down into units of meaning. The work of classifying the units of meaning is repeated by reading the content of the statements, marking the units of meaning.
**4th stage:** Transformation of the meanings of the description in a phenomenologically and psychologically sensitive way	At this stage, some of the participants’ original expressions are changed so that the psychological meaning of what the participants expressed can be grasped more directly. At this stage, the meaning units are classified according to similarity and then constituents and subconstituents are identified.
**5th stage:** Summarization of findings	Synthesis and integration of the revelations that emerged from the transcripts, and that are contained in the units of meaning. The researcher writes a new text in which they express, based on phenomenological concepts and terms, the learned structure, as well as the connections between the various units of meaning.

**Table 2:** Sociodemographic and professional data of participants (N = 16).

#	Age (Years)	Gender	Professional Experience (Years)	Working Place	Education	Specific Training in Palliative Care
P1	33	Female	12	Community Team/Home-Based Care	Bachelor	No
P2	47	Female	26	Community Team/Home-Based Care	Bachelor	Yes
P3	26	Female	4	Community Team/Home-Based Care	Master	Yes
P4	31	Female	9	Specialized Palliative Care Unit	Master	Yes
P5	55	Female	34	Community Team/Home Based Care	Bachelor	Yes
P6	34	Male	11	Specialized Palliative Care Unit	Master	No
P7	37	Female	14	Specialized Rehabilitation Unit	Master	No
P8	36	Male	15	Community Team/Home-Based Care	Master	Yes
P9	39	Female	18	Community Team/Home-Based Care	Master	Yes
P10	39	Male	14	Community Team/Home-Based Care	Bachelor	Yes
P11	35	Female	9	Specialized Rehabilitation Unit	Bachelor	No
P12	37	Female	14	Specialized Rehabilitation Unit	Bachelor	No
P13	30	Female	8	Specialized Rehabilitation Unit	Master	No
P14	34	Male	12	Community Team/Home-Based Care	Bachelor	Yes
P15	33	Female	11	Community Team/Home-Based Care	Bachelor	No
P16	43	Female	20	Specialized Rehabilitation Unit	Bachelor	No

**Table 3:** An overview of the constituents and subconstituents.

Constituents	Subconstituents
Undulating course of a complex disease	(a) Rapid decline and decreased functionality; (b) Non-acceptance of the disease and unrealistic expectations of a cure.
Barriers of person-centered care	(a) Lack of intra- and inter-professional communication; (b) Late diagnosis and referral to PC; (c) Shortage of human and technical resources; (d) Lack of specific training; (e) Caregiver burden; (f) Precarious socioeconomic conditions of families.
Enablers of person-centered care	(a) Collaborative teamwork; (b) Specialized support from associations supporting people with ALS; (c) Therapeutic relationship with the ill person and family.
Transition between curative and palliative care	(a) Access to narratives as a strategy for preserving human dignity; (b) Respect for wishes and preferences; (c) Seeking palliative care physiotherapy; (d) Offering psychosocial and spiritual support.

## Data Availability

All data generated or analyzed during this study are included in this article. This article is based on the first author’s master’s dissertation in Palliative Care at the School of Health Sciences—Polytechnic University of Leiria.

## Supplementary Materials

The following supporting information can be downloaded at: https://www.mdpi.com/article/10.3390/bs15040510/s1. Supplementary Table S1: Consolidated criteria for reporting qualitative studies (COREQ): 32-item checklist. Reference (Tong et al., 2007) is cited within the Supplementary Materials.
